# Elevating Haloperoxidase Expression in *Escherichia coli* through Fusion with a Formate Oxidase

**DOI:** 10.1002/cbic.70322

**Published:** 2026-04-08

**Authors:** Angelique Pothuizen, Jacob M. A. van Hengst, Ron Wever, Peter-Leon Hagedoorn, Frank Hollmann

**Affiliations:** ^1^ Department of Biotechnology Delft University of Technology Delft Netherlands; ^2^ Van ‘t Hoff Institute of Molecular Sciences University of Amsterdam Amsterdam Netherlands

**Keywords:** biocatalysis, formate oxidase, halogenation, haloperoxidase, protein fusion

## Abstract

Vanadium–dependent haloperoxidases (VHPOs) are attractive biocatalysts for halofunctionalisation chemistry, but their routine use is frequently constrained by poor soluble recombinant expression. Here, we explore protein fusion as a construct‐level strategy to simultaneously improve soluble expression of the vanadium chloroperoxidase from *Curvularia inaequalis* (*Ci*VCPO) and enable in situ H_2_O_2_ generation via formate oxidase from *Aspergillus oryzae* (*Ao*FOx). A panel of *Ao*FOx–*Ci*VCPO fusion designs was generated by varying enzyme orientation, linker length and linker architecture. Notably, fusion constructs displayed markedly increased haloperoxidase activity yields in crude lysates (up to ~9‐fold relative to non‐fused *Ci*VCPO), whereas *Ao*FOx activity decreased (approximately 36%–75%) compared to the individually expressed oxidase. A representative construct (*Ci*VCPO–10 aa flexible linker–*Ao*FOx) catalysed formate‐driven bromination of activated arenes (phenol, thymol) and oxidative bromolactonisation of 4‐pentenoic acid in crude extracts, giving product distributions consistent with hypobromite‐mediated reactivity. Time‐course experiments revealed that product formation was concentrated in the first 2 h and subsequently declined. H_2_O_2_‐spiking partially restored activity, and sustained turnover was observed in a hypohalite‐free sulfoxidation model reaction, implicating hypobromite‐mediated deactivation of the *Ao*FOx domain as a principal robustness‐limiting factor.

## Graphical abstract

Fusing *Aspergillus oryzae* formate oxidase (*Ao*FOx) to *Curvularia inaequalis* vanadium chloroperoxidase (*Ci*VCPO) boosts soluble expression and enables formate‐driven, in situ H_2_O_2_ supply for halogenation. Screening linker/orientation variants delivered up to 9‐fold higher haloperoxidase activity in *E. coli* lysates, supporting arene bromination and bromolactonisation. Time courses and H_2_O_2_ spiking reveal hypobromite‐induced *Ao*FOx deactivation as the main robustness limit.

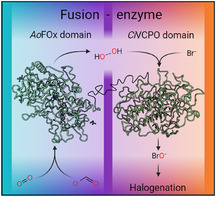

## 1 Introduction

**SCHEME 1:**
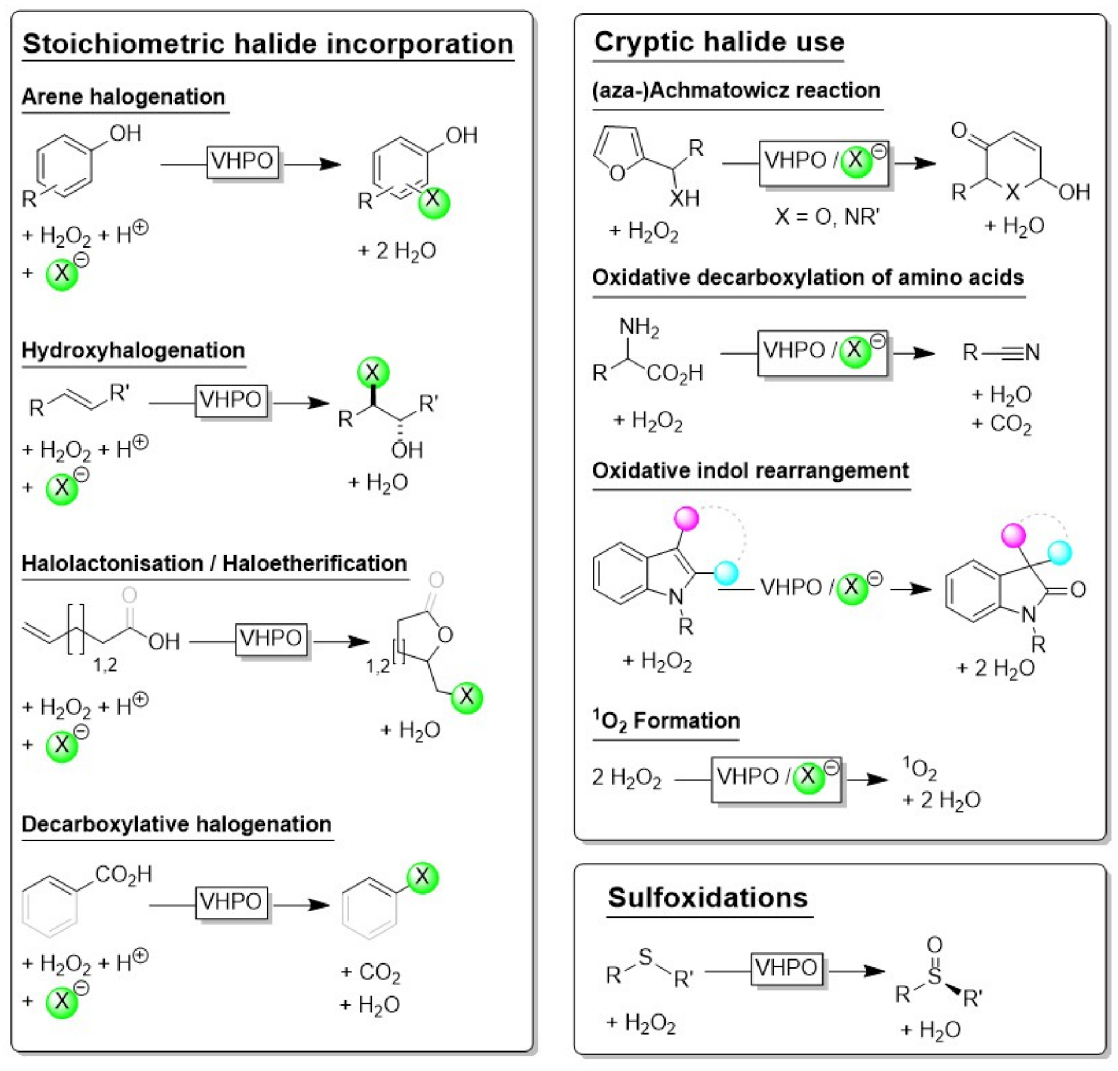
Selection of haloperoxidase‐catalysed oxidation reactions.

Haloperoxidases are enzymes that use hydrogen peroxide to oxidatively activate halide ions, generating the corresponding hypohalous acids [1]. These highly reactive species can subsequently react with a broad range of organic substrates, which makes haloperoxidases attractive and versatile biocatalysts for halofunctionalisation chemistry (Scheme 1) [2].

Early examples of haloperoxidase reactions i═nclude the halogenation of activated arenes, such as phenols [3, 4, 5, 6, 7], as well as other activated aromatic substrates [8, 9]. Subsequently, halohydroxylation of C═C double bonds [10, 11] and the mechanistically related halocyclisation reactions [12, 13, 14, 15], including their intermolecular variant [16], also gained prominence, alongside the decarboxylative halogenation of conjugated carboxylates [17, 18, 19, 20, 21]. N‐halogenation [22] and S‐halogenation [23] are also worth mentioning.

In addition to these direct halogenation reactions, ‘cryptic’ halogenations have been described, i.e., haloperoxidase‐catalysed oxidations that proceed via a transient halide‐derived intermediate which rearranges to the final product while releasing the halide again; in effect, the halide is used catalytically. Amongst these transformations, the (aza)Achmatowicz reaction [24, 25], oxidative amino acid decarboxylation to nitriles [26, 27], and the recently reported oxidative rearrangement of indoles [28] are particularly noteworthy. Cryptic haloperoxidase‐catalysed disproportionation of H_2_O_2_ to singlet oxygen [29] may also become attractive from a synthetic perspective.

Finally, the direct oxygenation of thioethers (in the absence of halides) [30] is worth noting, especially where enantioselectivity can be achieved.

Haloperoxidases are commonly grouped into two major classes: heme‐containing haloperoxidases and vanadium‐containing haloperoxidases (VHPOs) [2]. VHPOs contain orthovanadate (VO_4_^3-^) in the active site and form a peroxovanadate intermediate during turnover. VHPOs are generally more tolerant towards H_2_O_2_ than their heme‐containing counterparts. VHPOs are further categorised as chloro‐, bromo‐ or iodoperoxidases, depending on the most electronegative halide that can be oxidised [1]. Both vanadium bromoperoxidases and vanadium chloroperoxidases are widespread in nature.

Early haloperoxidase studies relied on enzyme isolation from native producers because robust heterologous expression platforms were not yet widely available. Although recombinant production is now feasible, the soluble expression of haloperoxidases remains challenging. In addition to process optimisation, solubility and activity can sometimes be improved through co‐expression of folding assistants (e.g., chaperones) that mitigate misfolding and aggregation [31]. Nevertheless, poor soluble yield continues to be a practical bottleneck for routine screening, engineering and application of VHPOs in whole‐cell and cell‐free biocatalysis.

Protein fusion offers a complementary, genetically encoded strategy to address solubility limitations at the construct‐design level. Solubility tags such as maltose‐binding protein and glutathione S‐transferase have long been used to increase the soluble fraction of aggregation‐prone recombinant proteins and to simplify purification [32, 33, 34]. Comparative studies of common fusion partners have shown that some tags, most notably SUMO and NusA, can provide particularly pronounced improvements in soluble expression for difficult targets, while also allowing efficient tag removal when required [35]. In parallel, engineered fluorescent fusion partners such as superfolder Green Fluorescent Protein (sfGFP) demonstrate how a robustly folding domain can stabilise otherwise poorly folding polypeptides; beyond enabling solubility enhancement, sfGFP also provides a convenient expression and folding readout for construct screening [36]. Recently, Yan et al. (2024) exploited this principle by fusing sfGFP to several unspecific peroxygenases (UPOs), improving expression in *E. coli* and supporting streamlined construct evaluation [37].

For multifunctional biocatalysts, fusion proteins can deliver additional benefits beyond acting as passive solubility enhancers. Genetic fusion fixes the stoichiometry of two activities in a single polypeptide, enables co‐purification, and can improve cascade performance by co‐localising enzymes, reducing intermediate loss, and, in favourable cases, supporting substrate channelling [38, 39]. The design of the fusion junction is critical: the relative domain order (N‐ versus C‐terminal positioning), choice of linker (flexible versus more rigid), and linker length can strongly influence folding, stability and catalytic function of each domain [40]. Consequently, practical fusion‐protein development often involves screening a small panel of linker architectures (e.g., Gly/Ser‐rich flexible linkers of varying length and/or semi‐rigid alternatives), alongside evaluation of domain orientation, to identify constructs that maximise soluble expression without compromising activity.

These considerations are particularly relevant for peroxidase‐driven catalysis because H_2_O_2_ is simultaneously the required oxidant and a potential stressor for both the host and the biocatalyst. Coupling a VHPO to an H_2_O_2_‐generating enzyme within a single fusion protein is therefore an attractive route to increase the local availability of H_2_O_2_ at the peroxidase domain and to reduce reliance on external H_2_O_2_ dosing.

In this work, we investigated the use of protein fusion to improve expression and performance of the well‐characterised VCPO from *Curvularia inaequalis* (*Ci*VCPO) [41, 42]. An appealing fusion partner is the formate oxidase from *Aspergillus oryzae* (*Ao*FOx), which produces H_2_O_2_ *in situ* by oxidising formate [43, 44, 45]. *Ao*FOx and *Ci*VCPO have previously been combined without genetic fusion to evaluate *Ao*FOx as an *in situ* H_2_O_2_ generation module, yielding promising results [46].

Here, we show that fusing these two fungal enzymes leads to a marked increase in soluble expression in *E. coli* relative to the individual enzymes.

## 2 Results and Discussion

### 2.1 Fusion Protein Construct Optimisation

To identify the optimal configuration of the *Ao*FOx–*Ci*VCPO fusion protein, variants were generated by varying: (i) linker length (5, 10 or 15 amino acids); (ii) linker flexibility (Gly/Ser/Ala‐rich for flexibility [47] or Pro‐rich for rigidity [48]); and (iii) enzyme orientation (the N‐ versus C‐terminal position of each enzyme in the fusion construct). Based on these parameters, expression plasmids for 12 fusion protein constructs were designed (Table 1), of which 10 were successfully generated.

**TABLE 1:** Overview of the designed system variations in order to find the optimal confirmation for the *Ao*FOx–*Ci*VCPO fusion protein. 6xHisTag = purification tag consisting of six Histidine residues.

Protein ‍name^a^	Fusion protein construct
A5fC	6xHisTag – *Ao*FOx – 5AA flexible linker ‐ *Ci*VCPO^b^
A5rC	6xHisTag – *Ao*FOx – 5AA rigid linker ‐ *Ci*VCPO^c^
C5fA	6xHisTag – *Ci*VCPO – 5AA flexible linker – *Ao*FOx^b^
C5rA	6xHisTag – *Ci*VCPO – 5AA rigid – *Ao*FOx^c^
A10fC	6xHisTag – *Ao*FOx – 10AA flexible linker – *Ci*VCPO^b^
A10rC	6xHisTag – *Ao*FOx – 10AA rigid linker – *Ci*VCPO^c^^,^ d
C10fA	6xHisTag – *Ci*VCPO – 10AA flexible linker – *Ao*FOx^b^
C10rA	6xHisTag – *Ci*VCPO – 10AA rigid linker – *Ao*FOx^c^
A15fC	6xHisTag – *Ao*FOx – 15AA flexible linker – *Ci*VCPO^c^^, ^ d
A15rC	6xHisTag – *Ao*FOx – 15AA rigid linker – *Ci*VCPO^c^
C15fA	6xHisTag – *Ci*VCPO – 15AA flexible linker – *Ao*FOx^b^
C15rA	6xHisTag – *Ci*VCPO – 15AA rigid linker – *Ao*FOx^c^

a Nomenclature: first letter N‐terminal enzyme (A for *Ao*FOx, C for *Ci*VCPO), number: length of the linker [N^o^ of amino acids], second letter: flexible (f) or rigid (r), last letter: C‐terminal enzyme (A for *Ao*FOx, C for *Ci*VCPO).

b Flexible protein linker sequence is adapted from Belsare et al. (2014) [47].

c Rigid protein linker sequence is adapted from Bakkes et al. (2019) [48].

d Expression plasmids for the fusion protein designs were designed but not successfully constructed.

Abbrevation: *Ao*FOx = *Aspergillus oryzae* Formate Oxidase, *Ci*VCPO = *Curvularia inaequalis* vanadium‐chloroperoxidase, AA = amino acid.

The 10 obtained expression plasmids were transformed into *E. coli* C43 (DE3) to produce the fusion proteins. Six out of the 10 constructs, all with *Ci*VCPO at the N‐terminus, showed evidence of overexpression by SDS–PAGE (Figure S1). The reason why constructs with N‐terminal *Ao*FOx exhibited poor expressibility is yet unknown to us. These six constructs were subsequently expressed in *E. coli* BL21 Gold (DE3), where five were successfully expressed (Figures S1–S4). For comparison of expression levels and specific activities, *Ao*FOx and *Ci*VCPO were also expressed individually in both *E. coli* strains.

Based on the SDS‐PAGE band intensities, *E. coli* BL21 Gold (DE3) produced higher expression levels of the fusion proteins and of non‐fused *Ao*FOx. In contrast, for non‐fused *Ci*VCPO no clear difference in expression was evident between the two E. coli strains from the SDS‐PAGE analysis. These differences can be explained by the expression characteristics for which each strain has been optimised. *E. coli* BL21 Gold (DE3) has been improved for heterologous protein expression (including by knockout of the OmpT protease) [49]. By comparison, *E. coli* C43 (DE3) is a *E. coli* BL21 (DE3) derivate selected for improved heterologous expression of toxic and membrane proteins [50]. As the designed fusion proteins are neither membrane proteins nor are they expected to be toxic to the host, its expression is likely to benefit primarily from the reduced protease activity in *E. coli* BL21 Gold (DE3).

**FIGURE 1:**
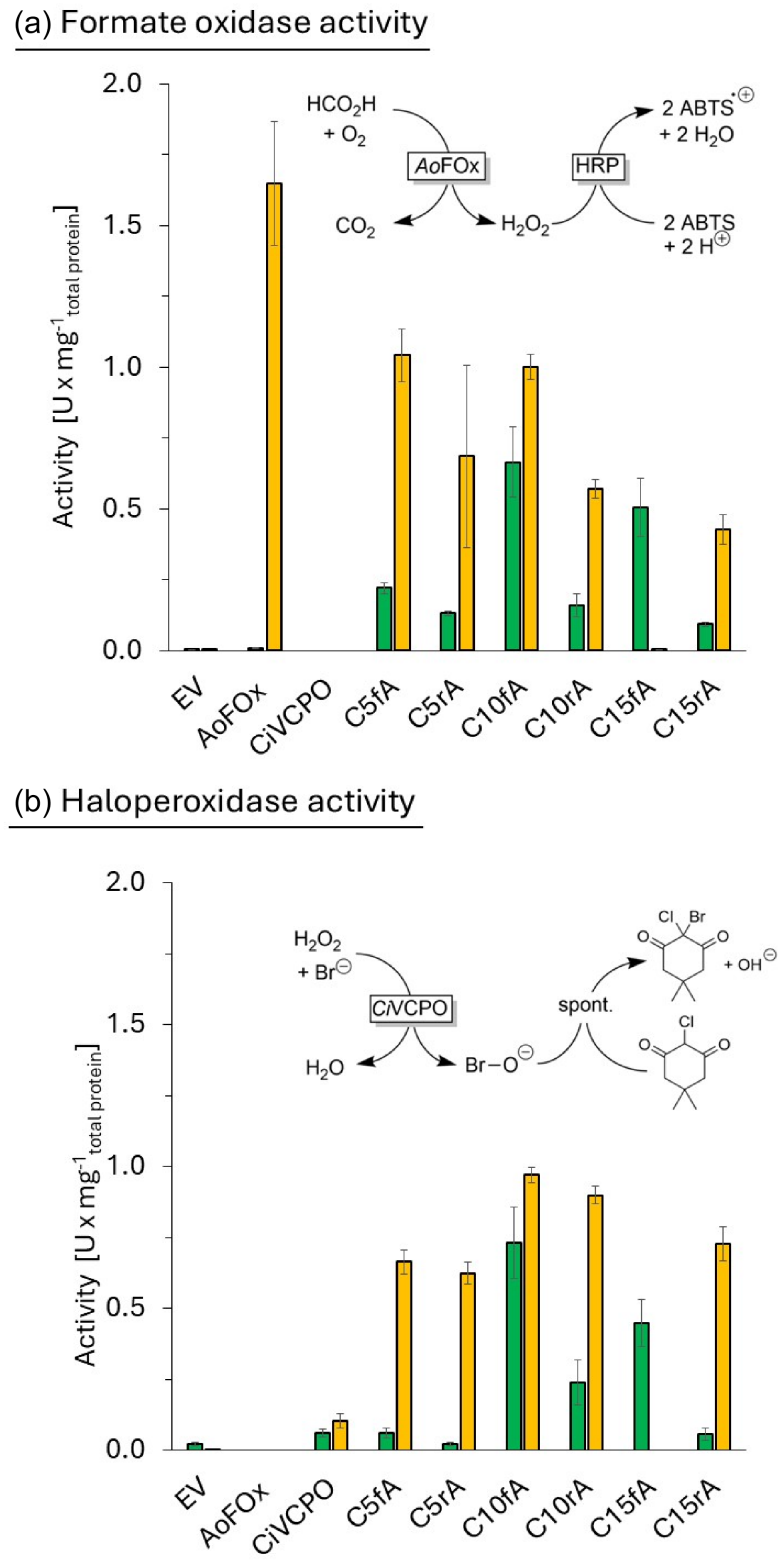
Specific activities of formate oxidase (a) and haloperoxidase activity (b) of some expressed fusion proteins (in crude cell lysates) compared to the non‐fused enzymes. Green: crude cell lysates from *E. coli* C43 (DE3), orange: crude cell lysates from *E. coli* BL21 Gold (DE3). Activity of *Ao*FOx was tested using an ABTS activity assay where the absorbance change at λ = 420 nm was traced. Reaction volume of the activity assay was 1 mL and a reaction contains 50 μL cell extract, 1 mM ABTS, and 10 U Horseradish Peroxidase in 50 mM acetate buffer (pH 4.5). Reactions were started with the addition of 50 mM sodium formate. *Ci*VCPO activity was measured using an MCD assay, absorbance at *λ* = 290 nm was traced. Reactions were performed in a 1 mL quartz cuvette, using a 1 mL reaction mixture consisting of 20 μL cell extract, 25 μM MCD, 5 mM KBr and 0.05 μM Na_3_VO_4_. Reactions were started with the addition of 5 mM H_2_O_2_. The values shown originate from technical triplicates (*n* = 3).

Next, we determined the activity yields of the individually expressed *Ao*FOx and *Ci*VCPO as well as of the fusion enzymes (Figure 1). Consistent with the qualitative findings of the SDS‐PAGE analysis, activity yields obtained in BL21 Gold (DE3) were higher than in C43 (DE3). Notably, the activity yield for non‐fused *Ao*FOx was approximately 160 times higher in BL21 Gold (DE3) than in C43 (DE3) (1.6 ± 0.22 U mg^−1^_total protein_ vs. 0.01 ± 0.0036 U mg^−1^_total protein_) while for non‐fused *Ci*VCPO this improvement was less pronounced (0.104 ± 0.025 U mg^−1^_total protein_ vs. 0.061 ± 0.015 U mg^−1^_total protein_).

In contrast, the activity yields for *Ao*FOx‐ and *Ci*VCPO activity in the fusion proteins were dramatically increased in case of *Ci*VCPO (up to 9‐fold) while in case of *Ao*FOx activity decreased by about 36% to 75% in the fusion proteins compared to the individual enzymes.

With the exception of C15fA, where practically no activity was observed in BL21 Gold (DE3), this strain generally yielded higher activity yields. The length and flexibility of the linker region had a less pronounced effect on the activity yield. A slightly higher activity in case of more flexible linkers can be attested. However, further detailed investigations into the kinetics of the individual constructs are required before any robust conclusions can be drawn.

### 2.2 The Fusion Protein Catalyses Formate‐Driven Halogenation Reactions

**FIGURE 2:**
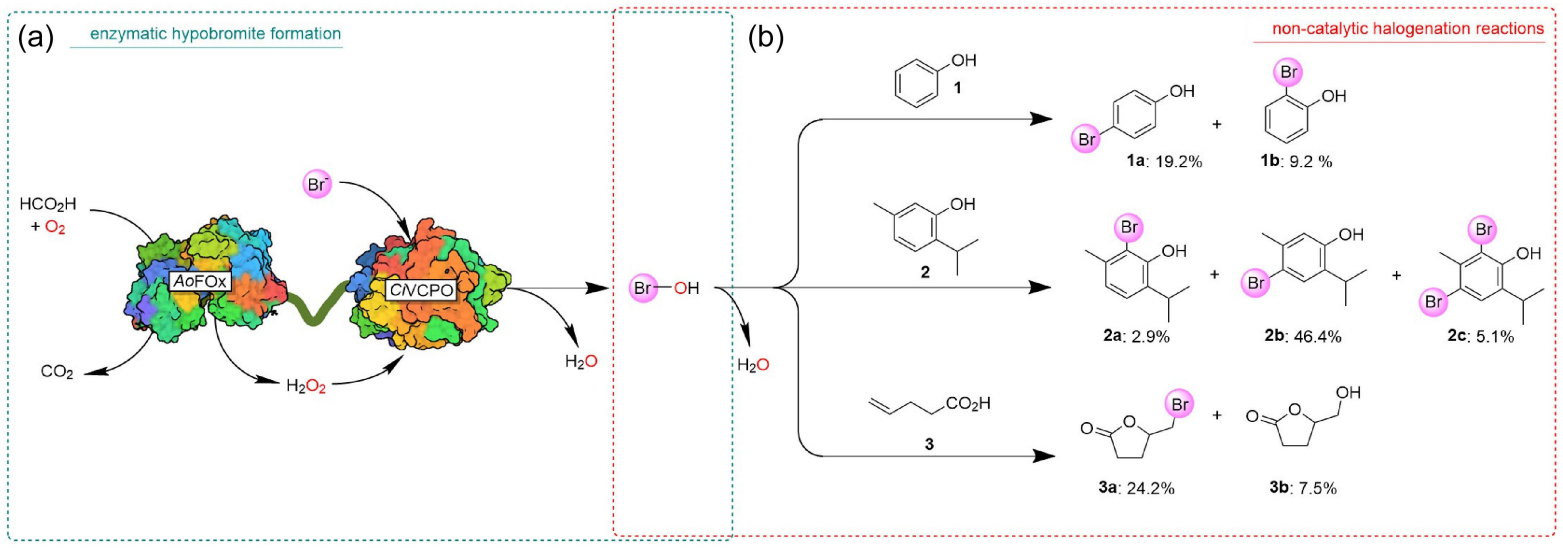
Validation of the dual function of the *Ci*VCPO–*Ao*FOx fusion enzyme constructs in representative transformations. (a) Fusion enzyme mediated generation of the hypobromite intermediate. (b) Non‐catalytic halogenation reaction of the organic substrates and the generated hypobromite. Reaction conditions: 200 mM acetate buffer (pH 4.0), 10 v% cosolvent (ACN), 1 mM substrate, and 10 v% crude cell extract (corresponding to 0.5 U *Ao*FOx activity). Formate (50 mM) and KBr (25 mM) were added every 2 h. Reactions were incubated at 30°C in an Eppendorf thermoshaker at 600 rpm. All concentrations given are final concentrations. Reported percentages are based on the ratios of the peak areas of the relevant compounds to that of the internal standard, as determined by GC analysis. Reactions were performed in duplicate.

To evaluate the catalytic activity of the proposed fusion proteins, we selected the fusion protein with a medium sized, flexible linker (C10fA). For this, phenol and thymol (representing activated arenes) and 4‐pentenoic acid as alkene were subjected to crude extracts of *E. coli* BL21 Gold (DE3) overexpressing C10fA (Figure 2).

In line with the diffusible character of hypbromite, the selectivities observed corresponded with the reactivities of the arene positions in phenol and thymol [5, 21]. *para*‐Bromination (with respect to the OH‐group) was preferred over *ortho*‐bromination while *meta*‐bromination was not observed. The more electron‐rich thymol gave higher conversions than phenol. With 4‐pentenoic acid, oxidative lactonisation was observed yielding the expected bromolactone (**3a**) together with the hydrolysis product (**3b**) [12, 13, 16].

It should, however, be noted that the majority of product formation occurred within the first 2 h, after which a pronounced decline was observed.

### 2.3 Deciphering the Robustness‐Limiting Factor

**FIGURE 3:**
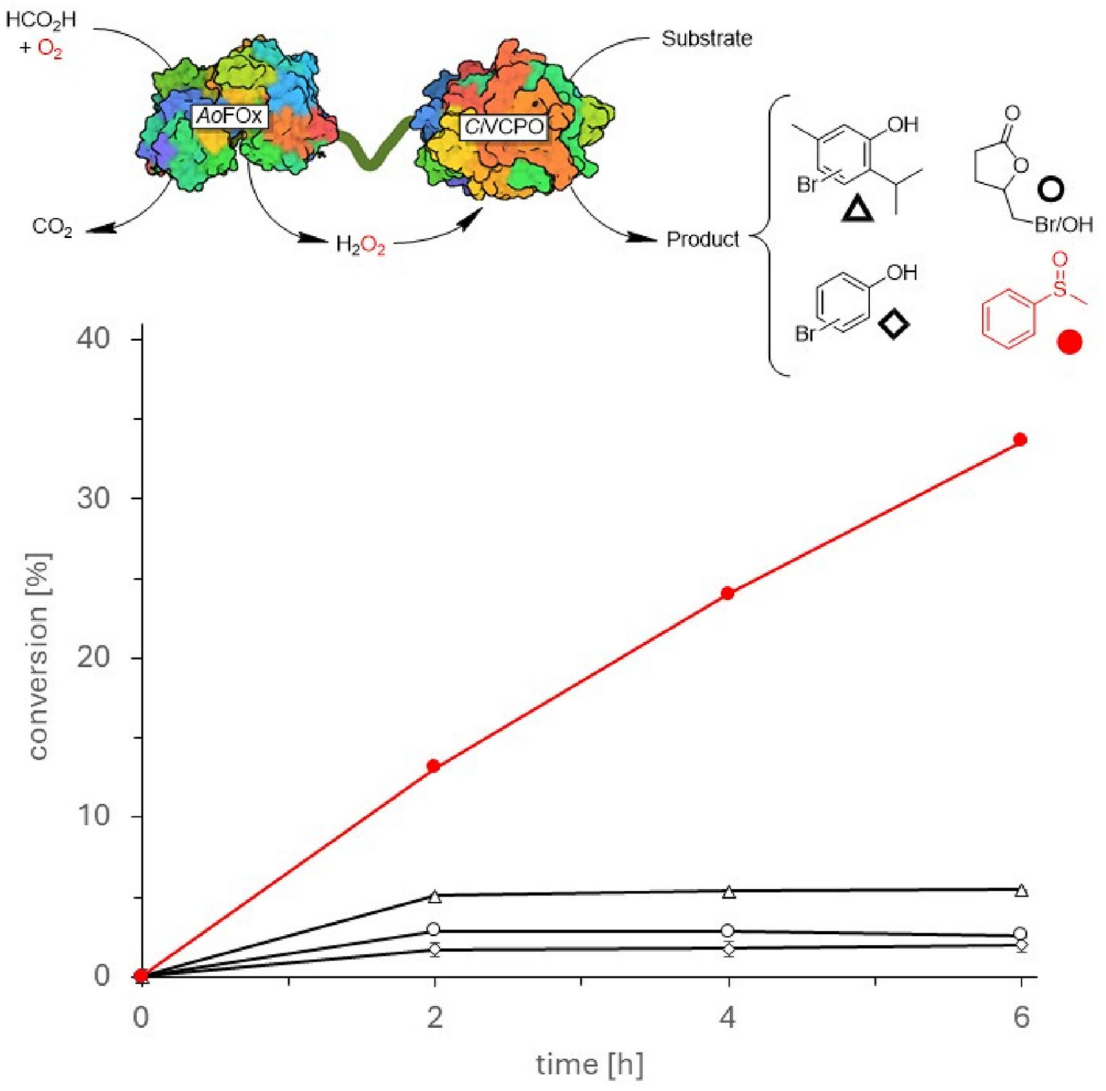
Representative time‐courses of halogenation reactions (black) and sulfoxidation (red) reactions catalysed by the fusion enzyme. Reaction conditions halogenation reactions: 200 mM acetate buffer (pH 4.0), 10 v% cosolvent (ACN), 1 mM substrate, and 10 v% crude cell extract (corresponding to 0.5 U *Ao*FOx activity). Formate (50 mM) and KBr (25 mM) were added to the reaction every 2 h. Reaction conditions sulfoxidation: 200 mM acetate buffer (pH 4.0), 10 v% cosolvent (ACN), 10 mM substrate, and 10 v% crude cell extract (corresponding to 0.5 U *Ao*FOx activity). Formate (50 mM) was added to the reaction every 2 h. All reaction mixtures were incubated at 30°C in an Eppendorf thermoshaker at 600 rpm. Values represent the ratios of the peak areas of the relevant compounds to that of the internal standard, as determined by GC analysis. Conversions were calculated based on the initial substrate concentration. Experiments were carried out as technical duplicates (*n* = 2), error bars are included.

An initial clue to the poor long‐term stability of the fusion‐protein‐catalysed system came from H_2_O_2_‐spiking experiments (Figure S5). Conversion of phenol and 4‐pentenoic acid partially resumed after addition of H_2_O_2_, indicating that the *Ao*FOx domain of the fusion protein becomes inactivated during catalytic turnover. Scott and co‐workers previously reported a comparable loss of activity and proposed that hypobromite deactivates the H_2_O_2_‐producing alcohol oxidase [51]. We therefore reasoned that hypobromite formation might likewise be the principal cause of the limited process robustness observed here, and evaluated sulfoxidation as a hypohalite‐free model reaction (Figure 3). Under these conditions, where hypobromite was absent, product formation was sustained for at least a 6 h reaction period. Collectively, these data support the hypothesis that hypobromite formation compromises the stability of the *Ao*FOx domain within the fusion protein. A possible contribution by a spontaneous background sulfoxidation was ruled out (Figure S6).

## 3 Conclusion

In this study, we extended the fusion‐enzyme concept to H_2_O_2_‐dependent oxidoreductases, using the *Ao*FOx–*Ci*VCPO system as a representative example. Notably, genetic fusion of two enzymes that are individually difficult to express resulted in a pronounced increase in soluble expression and, consequently, in overall activity yield. A deeper exploration of the parameter space (C vs. N terminus, spacer length and spacer rigidity) gaining statistical and structural insights may result in a better understanding of the underlying molecular reasons.

Preparative implementation of this catalyst system for halogenation reactions is currently limited by insufficient robustness of the H_2_O_2_‐generating *Ao*FOx module. Future work will focus on elucidating the underlying deactivation mechanism, including whether loss of activity arises from HOBr‐mediated oxidation of catalytically important residues and/or from halogenation of the flavin isoalloxazine moiety.

## 4 Experimental Section

### 4.1 Chemicals

All enzymes and reagents used for construction of the expression plasmids were purchased from New England Biolabs. The reference compound (*S*)‐γ‐hydroxymethyl‐α,β‐butenolide was synthesised from levoglucosenone according to Bonneau et al. [52] The synthesised (*S*)‐γ‐hydroxymethyl‐α,β‐butenolide was subsequently brominated according to Mattes and Benezra [53] as described in the Supporting Information. Unless otherwise stated, all other chemicals were purchased from Sigma‐Aldrich.

### 4.2 Construction of Fusion Protein Variants

Expression plasmids for the fusion protein variants were constructed in‐house. A detailed description of the plasmid design and cloning procedures is provided in the Supporting Information.

### 4.3 Production of Fusion Proteins

Fusion protein expression plasmids were transformed into chemically competent *E. coli* BL21 Gold (DE3) or *E. coli* C43 (DE3) cells. Recombinant strains were cultivated in Terrific Broth (TB; 12 g L^−1^ peptone, 24 g L^−1^ yeast extract, 4 mL L^−1^ 87% glycerol, 100 mM potassium phosphate (KPi) buffer, pH 7.0) for approximately 24 h. Expression cultures were inoculated to an optical density at 600 nm (OD_600_) of 0.05 using overnight pre‐cultures grown in Luria–Bertani (LB) medium (10 g L^−1^ peptone, 5 g L^−1^ yeast extract, 5 g L^−1^ NaCl). Cultures were incubated at 37°C and 180 rpm for 2.5–3 h until an OD_600_ of 0.5 was reached. Protein production was induced by addition of isopropyl β‐D‐1‐thiogalactopyranoside (IPTG; 0.5 mM final concentration). Following induction, the incubation temperature was reduced to 18°C for the remainder of the cultivation period (18‐20 hours).

### 4.4 Preparation of Crude Cell Extract

Cells expressing the proteins of interest were harvested by centrifugation at 17 500 × g for 30 min at 4°C. The supernatant was discarded and the pellets were washed with 50 mM Tris–H_2_SO_4_ buffer (pH 7.5). Cell pellets were resuspended in the same buffer to a concentration of 50 g L^−1^ (wet cell weight). Cells were disrupted in three consecutive passes using a CF1 Cell Disruptor (Constant Systems) at 1.5 kbar. The lysate was clarified by centrifugation at 30 000 × g for 30 min at 4°C to remove cell debris. The resulting cell‐free extract was stored at −20°C until further use.

### 4.5 SDS–PAGE Analysis

Whole‐cell SDS–PAGE samples were normalised to an OD_600_ of 0.5 in 1.0 mL. Cells were pelleted and resuspended in 71 μL Milli‐Q water, followed by addition of 25 μL 4×Laemmli sample buffer (Bio‐Rad) and 4 μL dithiothreitol (DTT; 100 mM). Crude cell extract samples were adjusted to a total protein concentration of 1 mg mL^−1^. All samples were denatured at 95°C for 3 min prior to loading. Bio‐Rad Precision Plus Protein Unstained Protein Standards (5 μL) were used as a molecular weight reference. Gels were run at a constant voltage of 200 V for 45 min.

### 4.6 ABTS Activity Assay for AoFOx

*Ao*FOx activity was determined using a spectrophotometric assay based on the oxidation of 2,2^′^‐azinobis‐(3‐ethylbenzothiazoline‐6‐sulphonic acid) (ABTS). Reactions (1.0 mL total volume) contained 50 mM acetate buffer (pH 4.5), crude cell extract (50 μL; diluted if necessary), ABTS (1 mM final concentration), and horseradish peroxidase (10 U; final amount per reaction). Reactions were initiated by addition of sodium formate (50 mM final concentration) from a 1 M stock solution. The change in absorbance at 420 nm was monitored for 90s using a Cary 60 UV–Vis spectrophotometer (Agilent Technologies). Enzyme activity was calculated from the initial linear rate using *ε*_420__nm_ = 36.0 mM^−1^ cm^−1^. All measurements were performed in triplicate.

### 4.7 MCD Activity Assay for CiVCPO

*Ci*VCPO activity was determined using a spectrophotometric assay based on monochlorodimedone (MCD) halogenation. Reactions (1.0 mL total volume) contained 100 mM citrate buffer, crude cell extract (20 μL), MCD (25 μM final concentration), KBr (5 mM final concentration), and Na_3_VO_4_ (0.05 μM final concentration). Reactions were initiated by addition of H_2_O_2_ (5 mM final concentration; 5 μL from a 1 M stock). The change in absorbance at 290 nm was monitored for 90s using a Cary 60 UV–Vis spectrophotometer. Enzyme activity was calculated from the initial linear rate using *ε*_290__nm_ = 19.9 mM^−1^ cm^−1^. All measurements were performed in triplicate.

### 4.8 Formate‐Driven Halogenation of Phenol, 4‐Pentenoic Acid and Thymol

Halogenation reactions were conducted in 2.0 mL Eppendorf tubes (500 μL total volume). Reactions contained 200 mM acetate buffer (pH 4.0), crude cell extract (50 μL), and substrate (1 mM final concentration). The crude cell extract volume used (50 μL) corresponded to 0.48 U *Ao*FOx activity and 0.50 U *Ci*VCPO activity. Substrates were dissolved in acetonitrile (ACN), which was used as a co‐solvent at 10% (v/v) final concentration. Sodium formate and KBr were added at 2 h intervals to final concentrations of 50 mM and 25 mM, respectively. As a negative control, crude cell extract from *E. coli* transformed with an empty‐vector (EV) plasmid was used. Reactions were incubated at 30°C and 600 rpm in an Eppendorf ThermoMixer for 6 h. Reactions were performed in duplicate. For experiments assessing potential *Ao*FOx inactivation reactions were initiated with sodium formate (50 mM final concentration). After 2 h, reactions were supplemented with H_2_O_2_ (2 mM final concentration) instead of sodium formate.

### 4.9 Formate‐Driven Oxidation of Thioanisole

Oxidation reactions were performed using the same conditions as the halogenation reactions, except that KBr was omitted. Reaction with thioanisole were performed in 2.0 mL glass reaction vials instead of the 2.0 mL plastic Eppendorf tube.

### 4.10 Gas Chromatography Analysis

Reaction samples were extracted with ethyl acetate containing an internal standard (5 mM) using a 1:1 (v/v) extraction ratio. GC analysis of the organic phase was performed on a Shimadzu GC‐2010 Plus system equipped with an AOC‐20i autosampler, an AOC‐20s carousel, an FID‐2010 Plus detector, and an Agilent CP‐Sil 8 CB column (25 m × 0.25 mm × 1.2 μm). Nitrogen was used as the carrier gas. Full analytical methods, including retention times for the relevant compounds, are provided in the Supporting Information.

## Supporting Information

The authors have cited additional references [54, 55, 56, 57] within the Supporting Information. Additional supporting information can be found online in the Supporting Information section. **Supporting Figure S1**: SDS‐PAGE analysis of fusion protein expression in *E.* *coli* C43 (DE3). Samples shown are whole cell SDS‐PAGE samples taken before (B.I.) and after (A.I.) induction of protein expression. The *Ci*VCPO and the *Ao*FOx have an expected molar weight of 61 kDa and 67 kDa, respectively. The expected molar weight of the fusion proteins is around 130 kDa. Bands corresponding to the designed fusion proteins (±130 kDa) are highlighted in the red boxes. Samples are normalized to a protein concentration of 1 mg/mL, based on the results of a BCA assay. **Supporting Figure S2**: SDS‐PAGE analysis of fusion protein expression in *E.* *coli* BL21‐Gold (DE3). Samples shown are whole cell SDS‐PAGE samples taken before (B.I.) and after (A.I.) induction of protein expression. The *Ci*VCPO and the *Ao*FOx have an expected molar weight of 61 kDa and 67 kDa, respectively. The expected molar weight of the fusion proteins is around 130 kDa. Bands corresponding to the designed fusion proteins (±130 kDa) are highlighted in the red boxes. Samples are normalized to a protein concentration of 1 mg/mL, based on the results of a BCA assay. **Supporting Figure S3**: SDS‐PAGE analysis of fusion protein expression in the crude cell extract of *E.* *coli* C43 (DE3). Bands corresponding to the designed fusion proteins (±130 kDa) are highlighted in the red boxes. Samples are normalized to a protein concentration of 1 mg/mL, based on the results of a BCA assay. **Supporting Figure S4**: SDS‐PAGE analysis of fusion protein expression in the crude cell extract of *E.* *coli* BL21 gold (DE3). Bands corresponding to the designed fusion proteins (±130 kDa) are highlighted in the red boxes. Samples are normalized to a protein concentration of 1 mg/mL, based on the results of a BCA assay. **Supporting Figure S5**: Comparison of total product formation in halogenation reactions using thymol, phenol, and 4‐pentenoic acid. The bars in red show total product formation (%) in bioconversion reactions where only sodium formate was used to drive the reaction. The green bars show total product formation (%) in the bioconversion reactions where the reactions were started with the addition of Formate, but after two hours H_2_O_2_ was added instead. **Supporting Supporting Figure S6**: Comparison of product formation during thioanisole oxidation reaction in presence of the fusion protein (a) and without fusion protein (b) and the chromatogram at t=0 (c). Reaction conditions: 200 mM acetate buffer (pH 4.0), 10 v% cosolvent (ACN), 10 mM substrate, and 10 v% crude cell extract (corresponding to 0.5 U *Ao*FOx activity for CE expression fusion protein C10fA). Reactions were incubated at 30 °C in an Eppendorf thermoshaker while shaking at 600 rpm. Reaction with the C10fA fusion protein were driven by adding formate (50 mM per two hours). To the negative control reaction with the EV construct, 2 mM H_2_O_2_ was added. Retention time thioanisole (substrate) = 4.1 minutes, retention time n‐dodecane (internal standard) = 4.9 min, retention time methyl‐phenyl‐sulfide (product) = 6.9 minutes. **Supporting Figure S7:** Structure prediction of fusion protein C10fA. Pink = 6xHisTag, green = haloperoxidase (CiVCPO), blue = protein linker (sequence P S P S T D Q S P S), red = Formate Oxidase (AoFOx). Structure is predicted using AlphaFold 3 webserver. Placement of the FAD cofactor, highlighted in yellow, in the *Ao*FOx domain of the fusion protein is based on the position of the FAD cofactor in the crystal structure of *Ao*FOx (PDB ID: 3Q9T). Placement of the Vanadate cofactor (VO_4_^‐^) in the haloperoxidase domain based on the available crystal structure of *Ci*VCPO (PDB ID: 1VNC). Amino Acids involved in binding of the cofactor and the catalytic mechanism are highlighted in cyan. **Supporting Table S1**: List of plasmids used in this study. **Supporting Table S2**: Primers used for generating the DNA fragments required for Gibson Assembly of the expression plasmids for all system variations of the *Ao*FOx – *Ci*VCPO fusion proteins. Underlined sequences show inserted 6xHisTag. **Supporting Table S3**: Description of DNA parts generated in this study to perform the Gibson Assembly of the 12 different fusion protein expression plasmids. GA = Gibson Assembly, AA = Amino Acid. **Supporting Table S4:** Expression plasmids for all designed system variations of the *Ao*FOx – *Ci*VCPO fusion proteins. Linker sequences are adapted from Belsare *et al.* (2014) or Bakkes *et al.* (2017). **Supporting Table S5**: Overview of analytical methods of GC analysis, including retention times of relevant compounds. All programs were run with a split ratio of 1:100 and a linear velocity of the carrier gas flow of 30 cm/sec.

## Funding

This study was supported by European Commission (Grant 101054658).

## Conflicts of Interest

The authors declare no conflicts of interest.

## Supporting information

Supplementary Material

## Data Availability

The data that support the findings of this study are openly available in 4TU at https://data.4tu.nl/, reference number 10.4121/74f18a79‐4210‐40f9‐9abd‐b896f02017de.
