# Supplementary material for: Elevating Haloperoxidase Expression in Escherichia coli through Fusion with a Formate Oxidase
Source: Chembiochem. 2026 Apr 8;27(7):e70322. doi: 10.1002/cbic.70322 (PMC13059055; doi:10.1002/cbic.70322)
Supplement: Supplementary file 1 — Supplementary Material [file CBIC-27-e70322-s001.pdf]

Supporting Information

Elevating Enzyme Performance through Protein Fusion of *Ao*Fox and *Ci*VCPO

Angelique Pothuizen[1], Jacob van Hengst[1], Ron Wever[2], Peter-Leon Hagedoorn[1] and Frank Hollmann[1]*

[1] Department of Biotechnology, Delft University of Technology, van der Maasweg 9, 2629HZ Delft, The Netherlands; e-mail: a.pothuizen@tudelft.nl

[2] Van 't Hoff Institute for Molecular Sciences, University of Amsterdam, Science Park 904, 1098 XH, Amsterdam, The Netherlands

Synthesis of (S)-γ-hydroxymethyl-α,β-butenolide and 5-(bromomethyl)dihydrofuran-2(*3H*)-one

Compounds (S)-γ-hydroxymethyl-α,β-butenolide and 5-(bromomethyl)dihydrofuran-2(*3H*)-one were synthesized to be used as references compounds for the GC analysis of the oxidative lactonization reaction. (S)-γ-hydroxymethyl-α,β-butenolide was synthesized from dihydrolevoglucosenone as described previously by Bonneau *et al*.[1]

Dihydrolevoglucosenone (1.03 mL, 10 mmol) and hydrogen peroxide (2.5 eq, 1.42 mL of a 50% solution in water) were mixed at 0 ºC. Cooling was removed and the reaction mixture was heated to 50 ºC. After TLC indicated full conversion, the reaction was quenched with 35 mmol $NaHSO_3$. The resulting solution was diluted with water and extracted three times with ethyl acetate. The combined organic phases were dried with $MgSO_4$ and concentrated under reduced pressure. Because a large amount of product was still in the aqueous phase, the aqueous phase was also concentrated, and the salts were washed multiple times with acetone. The acetone was removed under vacuum and the residue was added to the residue from the previous extraction. Silica chromatography (30% → 50% acetone in pentane) yields the title compound as colourless oil (820 mg, 71%). ^{1}H NMR (400 MHz, $CDCl_3$) δ 4.65 (dddd, *J* = 7.6, 6.7, 4.6, 2.9 Hz, 1H), 3.90 (dd, *J* = 12.5, 2.9 Hz, 1H), 3.66 (dd, *J* = 12.5, 4.6 Hz, 1H), 3.19 (s, 1H), 2.68 – 2.49 (m, 2H), 2.28 (dddd, *J* = 12.8, 9.8, 7.6, 5.9 Hz, 1H), 2.15 (dddd, *J* = 12.9, 10.0, 8.0, 6.7 Hz, 1H); ^{13}C NMR (101 MHz, $CDCl_3$) δ 178.1, 81.0, 64.1, 28.8, 23.2.

(S)-γ-hydroxymethyl-α,β-butenolide was brominated according to the procedure by Mattes and Berenza.[2]

(S)-γ-hydroxymethyl-α,β-butenolide (0.50 g, 4.31 mmol) and CBr_4 (1.2 eq,1.71 g) were dissolved in 10 mL acetonitrile and cooled to 0 ºC after which Ph_3P (1.2 eq,1.36 g) was added in small portions. The reaction mixture was allowed to warm to room temperature overnight, after which a scoop of celite was added and the reaction mixture was concentrated under reduced pressure. The residue was purified with silica chromatography (30% EtOAc in pentane) yielding the title compound as a yellowish oil (156 mg, 20%); ^{1}H NMR (400 MHz, $CDCl_3$) δ 4.76 (dddd, *J* = 7.3, 6.5, 5.5, 4.6 Hz, 1H), 3.60 – 3.57 (m, 1H), 3.57 – 3.52 (m, 1H), 2.73 – 2.52 (m, 2H), 2.45 (dddd, *J* = 13.1, 9.8, 7.3, 5.6 Hz, 1H), 2.13 (dddd, *J* = 13.1, 10.2, 8.0, 6.6 Hz, 1H); 13C NMR (101 MHz, CDCl3) δ 176.3, 77.9, 34.2, 28.4, 26.2.

Cloning information for construction of expression plasmids for fusion protein variants

As the expression plasmids for Formate Oxidase from *Aspergillus oryzae* (*Ao*FOx) and the Vanadium-dependent ChloroPeroxidase from *Curvularia inaequalis* (*Ci*VCPO) were already available in-house, they were used as starting points for construction of the fusion protein expression plasmids. To generate the fusion proteins, the stop codon was removed from sequence encoding the protein in the N-terminal position of the fusion protein. After the last codon of the N-terminal protein, the linker sequence was inserted, followed by the coding sequence for the C-terminal protein. Table S1 shows a list of plasmids used in this study, with the exception of V_*Ao*FOx, V_*Ci*VCPO, and pET_EV, all plasmids were constructed in house during this study.

Table S1: List of plasmids used in this study

Plasmid name	Backbone	Genetic information
**V_*Ao*FOx**	pET21c(+)	*Ao*FOx (not codon optimized) with N-terminal 6x-HisTag
**V_*Ci*VCPO**	pBADgIIIB	*Ci*VCPO (not codon optimized)
**pET_EV**	pET24b	pET24b vector with an empty MCS
**V_A5fC**	pET21c(+)	*Ao*FOx fused to *Ci*VCPO with a 5AA flexible protein linker and a N-terminal 6His-Tag
**V_A5rC**	pET21c(+)	*Ao*FOx fused to *Ci*VCPO with a 5AA rigid protein linker and a N-terminal 6His-Tag
**V_C5fA**	pET21c(+)	*Ci*VCPO fused to *Ao*FOx with a 5AA flexible protein linker and a N-terminal 6His-Tag
**V_C5rA**	pET21c(+)	*Ci*VCPO fused to *Ao*FOx with a 5AA Rigid protein linker and a N-terminal 6His-Tag
**V_A10fC**	pET21c(+)	*Ao*FOx fused to *Ci*VCPO with a 10AA flexible protein linker and a N-terminal 6His-Tag
**V_C10fA**	pET21c(+)	*Ci*VCPO fused to *Ao*FOx with a 10AA flexible protein linker and a N-terminal 6His-Tag
**V_C10rA**	pET21c(+)	*Ci*VCPO fused to *Ao*FOx with a 10AA rigid protein linker and a N-terminal 6His-Tag
**V_A15rC**	pET21c(+)	*Ao*FOx fused to *Ci*VCPO with a 15AA rigid protein linker and a N-terminal 6His-Tag
**V_C15fA**	pET21c(+)	*Ci*VCPO fused to *Ao*FOx with a 15AA flexible protein linker and a N-terminal 6His-Tag
**V_C15rA**	pET21c(+)	*Ci*VCPO fused to *Ao*FOx with a 15AA rigid protein linker and a N-terminal 6His-Tag

Table S2 provides a list of primers used in this work. These primers were used to perform the PCRs required to produce the DNA fragments that will be used to construct the fusion protein expression plasmids using Gibson Assembly. Table S3 shows a description of all designed DNA fragments and the used primer combinations for generation of each fragment.

Table S2: Primers used for generating the DNA fragments required for Gibson Assembly of the expression plasmids for all system variations of the *Ao*FOx – *Ci*VCPO fusion proteins. Underlined sequences show inserted 6xHisTag.

Primer name	Primer Sequence (5' → 3')
**P_01**	GTGGTGGTGGTGGTGGTGCATATGTATATCTCCTTCTTAAAG
**P_02**	GATCCGGCTGCTAACAAAGCCC
**P_03**	ATGCACCACCACCACCACCACGCAACCGATGGTAGCCATTTTG
**P_04**	GGTGCTCGGGCTCGGCTCGAGTGCGGCCGCATAC
**P_05**	GCTCGGGCTCTGATCGGTGCTCGGGCTCGGCTCGAGTGCGGCCGCATAC
**P_06**	CCGAGCCCGAGCACCATGGGGTCCGTTACACCCATC
**P_07**	CTTTGTTAGCAGCCGGATCCTACGGCGCCTCCTTGACTAC
**P_08**	CCGAGCCCGAGCACCGATCAGAGCCCGAGCATGGGGTCCGTTACACCCATC
**P_09**	GATCAGAGCCCGAGCACCGGTGATGCGGTGATGGGGTCCGTTACACCCATC
**P_10**	CGGCGGCGGCGGTTCCTCGAGTGCGGCCGCATAC
**P_11**	TGGTGGTGGTGGCTCCGGCGGCGGCGGTTCCTCGAGTGCGGCCGCATAC
**P_12**	GAACCGCCGCCGCCGATGGGGTCCGTTACACCCATC
**P_13**	GAACCGCCGCCGCCGGAGCCACCACCACCAATGGGGTCCGTTACACCCATC
**P_14**	GAGCCACCACCACCACTGCCGCCGCCGCCGATGGGGTCCGTTACACCCATC
**P_15**	ATGCACCACCACCACCACCACGGGTCCGTTACACCCATCC
**P_16**	GGTGCTCGGGCTCGGCGGCGCCTCCTTGACTAC
**P_17**	GCTCGGGCTCTGATCGGTGCTCGGGCTCGGCGGCGCCTCCTTGACTAC
**P_18**	CCGAGCCCGAGCACCATGGCAACCGATGGTAGCCATTTTG
**P_19**	CTTTGTTAGCAGCCGGATCTCACTCGAGTGCGGCCGCATACAG
**P_20**	CCGAGCCCGAGCACCGATCAGAGCCCGAGCATGGCAACCGATGGTAGCCATTTTG
**P_21**	GATCAGAGCCCGAGCACCGGTGATGCGGTGATGGCAACCGATGGTAGCCATTTTG
**P_22**	CGGCGGCGGCGGTTCCGGCGCCTCCTTGACTAC

**P_23**	TGGTGGTGGTGGCTCCGGCGGCGGCGGTTCCGGCGCCTCCTTGACTAC
**P_24**	GAACCGCCGCCGCCGATGGCAACCGATGGTAGCCATTTTG
**P_25**	GAACCGCCGCCGCCGGAGCCACCACCACCAATGGCAACCGATGGTAGCCATTTTG
**P_26**	GAGCCACCACCACCACTGCCGCCGCCGCCGATGGCAACCGATGGTAGCCATTTTG

Table S3: Description of DNA parts generated in this study to perform the Gibson Assembly of the 12 different fusion protein expression plasmids. GA = Gibson Assembly, AA = Amino Acid

DNA fragment	Forward Primer	Reverse Primer	Template	Description DNA fragment for Gibson Assembly
**DNA_01**	P_02	P_01	V_*Ao*FOx	pET21c(+) linear backbone. Contains start codon + N-terminal 6HisTag
**DNA_02**	P_03	P_04	V_*Ao*FOx	AoFOx with N-terminal 6HisTag and 5AA flexible linker extension for GA
**DNA_03**	P_03	P_05	V_*Ao*FOx	AoFOx with N-terminal 6HisTag and 10AA flexible linker extension for GA
**DNA_04**	P_06	P_07	V_*Ci*VCPO	CiVCPO with 5AA flexible linker extension and pET21c(+) overlap for GA
**DNA_05**	P_08	P_07	V_*Ci*VCPO	CiVCPO with 10AA flexible linker extension and pET21c(+) overlap for GA
**DNA_06**	P_09	P_07	V_*Ci*VCPO	CiVCPO with 15AA flexible linker extension and pET21c(+) overlap for GA
**DNA_07**	P_03	P_10	V_*Ao*FOx	AoFOx with N-terminal 6HisTag and 5AA rigid linker extension for GA
**DNA_08**	P_03	P_11	V_*Ao*FOx	AoFOx with N-terminal 6HisTag and 10AA rigid linker extension for GA
**DNA_09**	P_12	P_07	V_*Ci*VCPO	CiVCPO with extension for 5AA rigid linker and pET21c(+) overlap for GA
**DNA_10**	P_13	P_07	V_*Ci*VCPO	CiVCPO with extension for 10AA rigid linker and pET21c(+) overlap for GA
**DNA_11**	P_14	P_07	V_*Ci*VCPO	CiVCPO with extension for 15AA rigid linker and pET21c(+) overlap for GA
**DNA_12**	P_15	P_16	V_*Ci*VCPO	CiVCPO with 6HisTag (N-terminus) and 5AA P-link extension for GA
**DNA_13**	P_15	P_17	V_*Ci*VCPO	CiVCPO with 6HisTag (N-terminus) and 10AA P-link extension for GA
**DNA_14**	P_18	P_19	V_*Ao*FOx	AoFOx with 5AA P-link extension and pET21c(+) overlap for GA
**DNA_15**	P_20	P_19	V_*Ao*FOx	AoFOx with 10AA P-link extension for 10AA P-link and pET21c(+) overlap for GA
**DNA_16**	P_21	P_19	V_*Ao*FOx	AoFOx with 10AA P-link extension for 15AA P-link and pET21c(+) overlap for GA
**DNA_17**	P_15	P_22	V_*Ci*VCPO	CiVCPO with 6HisTag (N-terminus) and 5AA rigid link extension for GA
**DNA_18**	P_15	P_23	V_*Ci*VCPO	CiVCPO with 6HisTag (N-terminus) and 10AA rigid link extension for GA
**DNA_19**	P_24	P_19	V_*Ao*FOx	AoFOx with 5AA rigid link extension and pET21c(+) overlap for GA
**DNA_20**	P_25	P_19	V_*Ao*FOx	AoFOx with 10AA rigid link extension for 10AA P-link and pET21c(+) overlap for GA
**DNA_21**	P_26	P_19	V_*Ao*FOx	AoFOx with 10AA rigid link extension for 15AA P-link and pET21c(+) overlap for GA

Prior to performing the Gibson Assembly reactions, all the PCR products were purified using a Monarch PCR and DNA cleanup kit *(T1030)* from New England Biolabs. An overview of the combinations of the Gibson Assembly parts to construct all 12 expression plasmid variations is given in Table S4. Gibson Assembly reactions were performed using the NEBuilder® HiFi DNA Assembly Master Mix from New England Biolabs, according to the manufacturer's instructions.

Table S4: Expression plasmids for all designed system variations of the *Ao*FOx – *Ci*VCPO fusion proteins. Linker sequences are adapted from Belsare *et al.* (2014) or Bakkes *et al.* (2017).[3,4]

Plasmid Name	Fusion Protein construct	DNA fragments used for Gibson Assembly			Linker sequence (Amino Acids)
**V_A5fC**	6HisTag - FOx - flexible linker (5AA) - VCPO	DNA_01	DNA_02	DNA_04	P S P S T [3]
**V_A5rC**	6HisTag - FOx - rigid linker (5AA) - VCPO	DNA_01	DNA_07	DNA_09	E P P P P[4]
**V_C5fA**	6HisTag - VCPO - flexible linker (5AA) - FOx	DNA_01	DNA_12	DNA_14	P S P S T[3]
**V_C5rA**	6HisTag - VCPO - rigid linker (5AA) - FOx	DNA_01	DNA_17	DNA_19	E P P P P[4]
**V_A10fC**	6HisTag - FOx - flexible linker (10AA) - VCPO	DNA_01	DNA_03	DNA_05	P S P S T D Q S P S[1]
**V_A10rC**	6HisTag - FOx - rigid linker (10AA) - VCPO	DNA_01	DNA_08	DNA_10	E P P P P L P P P P [4]
**V_C10fA**	6HisTag - VCPO - flexible linker (10AA) - FOx	DNA_01	DNA_13	DNA_15	P S P S T D Q S P S[3]
**V_C10rA**	6HisTag - VCPO - rigid linker (10AA) - FOx	DNA_01	DNA_18	DNA_20	E P P P P L P P P P[4]
**V_A15fC**	6HisTag - FOx - flexible linker (15AA) - VCPO	DNA_01	DNA_03	DNA_06	P S P S T D Q S P S T G D A V[3]
**V_A15rC**	6HisTag - FOx - rigid linker (15AA) - VCPO	DNA_01	DNA_08	DNA_11	E P P P P E P P P P L P P P P[4]
**V_C15fA**	6HisTag - VCPO - flexible linker (15AA) - FOx	DNA_01	DNA_13	DNA_16	P S P S T D Q S P S T G D A V[3]
**V_C15rA**	6HisTag - VCPO - rigid linker (15AA) - FOx	DNA_01	DNA_18	DNA_21	E P P P P E P P P P L P P P P[4]

PCR cycler programs

All PCR reaction were performed using the Q5 2x Master Mix polymerase form New England Biolabs, according to the instruction of the manufacturers. To construct the different part for the Gibson assembly reactions, two different PCR cycler programs were used.

<u>Cycler program 1:</u> Used for fragments DNA_01 and DNA_02

- Initial denaturation: 30 seconds, 98 ℃
- Cycling program: 30 cycles
    - Denaturation: 10 seconds, 98 ℃
    - Annealing: 20 seconds, 65 ℃
    - Extension: 3 minutes, 72 ℃
- Final extension: 5 minutes, 72 ℃

<u>PCR cycler program 2:</u> Used for generation of fragments DNA_03 - DNA_21

- Initial denaturation: 30 seconds, 98℃
- Cycling program: 30 cycles
    - Denaturation: 10 seconds, 98 ℃
    - Annealing: 20 seconds, 70 ℃
    - Extension: 1 minute, 72 ℃
- Final extension: 5 minutes, 72 ℃

SDS-PAGE analysis of whole cell samples of *E. coli* C43 (DE3) and *E. coli* BL21 gold (DE3)

Figures S1 and S2 show the analysis of the whole cell SDS-PAGE samples taken before (B.I.) and after (A.I.) induction of protein expression for *E. coli* strains C43(DE3) and BL21-Gold(DE3) respectively. Expected protein sizes are 67 kDa for the *Ci*VCPO, and 61 kDa for the *Ao*FOx.[5,6] The resulting fusion protein should have a size of 128 kDa.

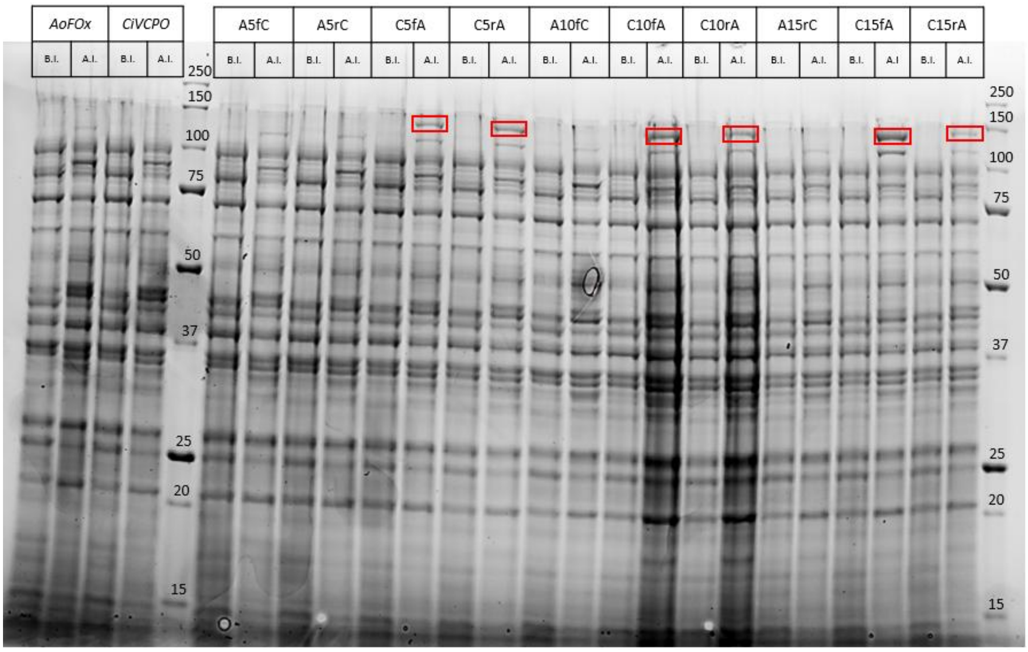

Figure S1: SDS-PAGE analysis of fusion protein expression in *E. coli* C43 (DE3). Samples shown are whole cell SDS-PAGE samples taken before (B.I.) and after (A.I.) induction of protein expression. The *Ci*VCPO and the *Ao*FOx have an expected molar weight of 61 kDa and 67 kDa, respectively. The expected molar weight of the fusion proteins is around 130 kDa. Bands corresponding to the designed fusion proteins (±130 kDa) are highlighted in the red boxes. Samples are normalized to a protein concentration of 1 mg/mL, based on the results of a BCA assay.

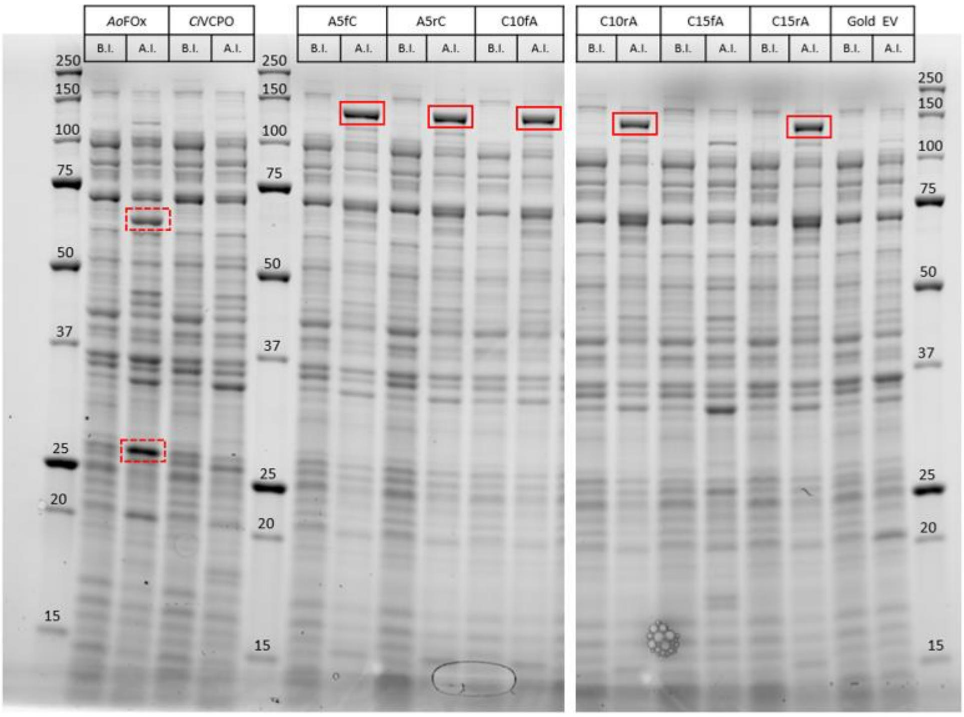

Figure S2: SDS-PAGE analysis of fusion protein expression in *E. coli* BL21-Gold (DE3). Samples shown are whole cell SDS-PAGE samples taken before (B.I.) and after (A.I.) induction of protein expression. The *Ci*VCPO and the *Ao*FOx have an expected

molar weight of 61 kDa and 67 kDa, respectively. The expected molar weight of the fusion proteins is around 130 kDa. Bands corresponding to the designed fusion proteins (±130 kDa) are highlighted in the red boxes. Samples are normalized to a protein concentration of 1 mg/mL, based on the results of a BCA assay.

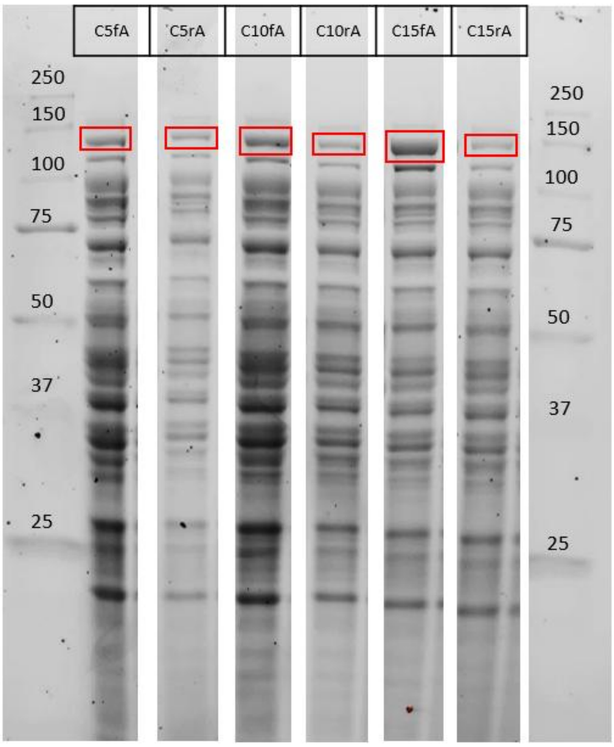

Figure S3: SDS-PAGE analysis of fusion protein expression in the crude cell extract of *E. coli* C43 (DE3). Bands corresponding to the designed fusion proteins (±130 kDa) are highlighted in the red boxes. Samples are normalized to a protein concentration of 1 mg/mL, based on the results of a BCA assay.

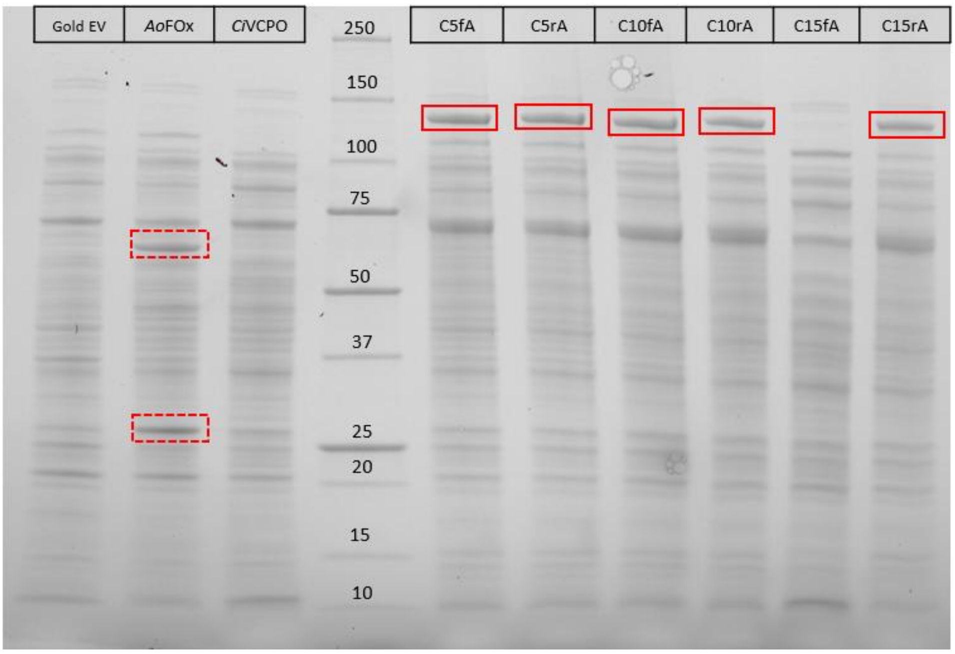

Figure S4: SDS-PAGE analysis of fusion protein expression in the crude cell extract of *E. coli* BL21 gold (DE3). Bands corresponding to the designed fusion proteins (±130 kDa) are highlighted in the red boxes. Samples are normalized to a protein concentration of 1 mg/mL, based on the results of a BCA assay.

GC programs and retention times

Bioconversion reactions were extracted with a 1:1 volumetric ratio of ethyl acetate containing 5mM of either n-dodecane or 1-octanol as internal standard. All samples were analysed using a Shimadzu GC-2010 Plus system equipped with an AOC-20i autosampler, an AOC-20s carousel, an FID-2010 Plus detector, and an Agilent CP-Sil 8 CB column (25 m × 0.25 mm × 1.2 µm), with N_2 as carrier gas. Table S5 shows the temperature profiles used to analyse the different reactions and includes retention times of all relevant compounds.

Table S5: Overview of analytical methods of GC analysis, including retention times of relevant compounds. All programs were run with a split ratio of 1:100 and a linear velocity of the carrier gas flow of 30 cm/sec.

Column	Temperature profile	Retention times
GC<br>CP-sil 8 CB (Agilent)<br>(25m x 0.25mm x 1.2 µm)<br>carrier gas: N_2	200 °C, hold 3.5 min<br>30 °C / min to 230 °C, hold 2.5 min<br>30 °C / min to 290 °C, hold 1.5 min<br>30 °C / min to 345 °C, hold 1 min	Phenol: 3.3 min<br>n-Dodecane (IS): 4.5 min<br>4-bromophenol: 5.5 min<br>2,4-dibromophenol: 6.7 min<br>2,4,6-tribromophenol: 10.0 min
GC<br>CP-sil 8 CB (Agilent)<br>(25m x 0.25mm x 1.2 µm)<br>carrier gas: N_2	100 °C, hold 1 min<br>30 °C / min to 180 °C, hold 0.6 min<br>30 °C / min to 220 °C, hold 3 min<br>30 °C / min to 345 °C, hold 1 min	4-pentenoic acid: 4.0 min<br>1-Octanol (IS): 5.8 min<br>γ-lactone: 7.0 min<br>Bromolactone: 7.9 min
GC<br>CP-sil 8 CB (Agilent)<br>(25m x 0.25mm x 1.2 µm)<br>carrier gas: N_2	240 °C, hold 5 min<br>20 °C / min to 345 °C, hold 1 min	n-Dodecane (IS): 2.6 min<br>Thymol: 3.0 min<br>Product 1: 4.1 min<br>Product 2: 5.2 min<br>Product 3: 6.6 min
GC<br>CP-sil 8 CB (Agilent)<br>(25m x 0.25mm x 1.2 µm)<br>carrier gas: N_2	180 °C, hold 5 min<br>30 °C / min to 240 °C, hold 2 min<br>30 °C / min to 345 °C, hold 1min	Thioanisole: 4.1 min<br>n-Dodecane (IS): 4.9 min<br>methyl-phenyl-sulfoxide: 6.9 min<br>methyl-phenyl-sulfone: 7.6 min

H_2O_2 spiking experiments to verify *Ao*FOx inactivation hypothesis

To confirm that *Ao*FOx stability indeed was the limiting factor of the reactions catalysed by the fusion protein, a set of experiments was performed where the reactions were started with the addition of formate. After the first two hours of the reaction, the reaction was supplied with H_2O_2 instead of more formate. Figure S5 compares the results of product formation in reactions only using Formate (red), and reactions that were started with formate but were supplied with H_2O_2 after the initial 2 hours had passed (green). For both phenol and 4-pentenoic acid, the addition of H_2O_2 indeed leads to higher product formation.

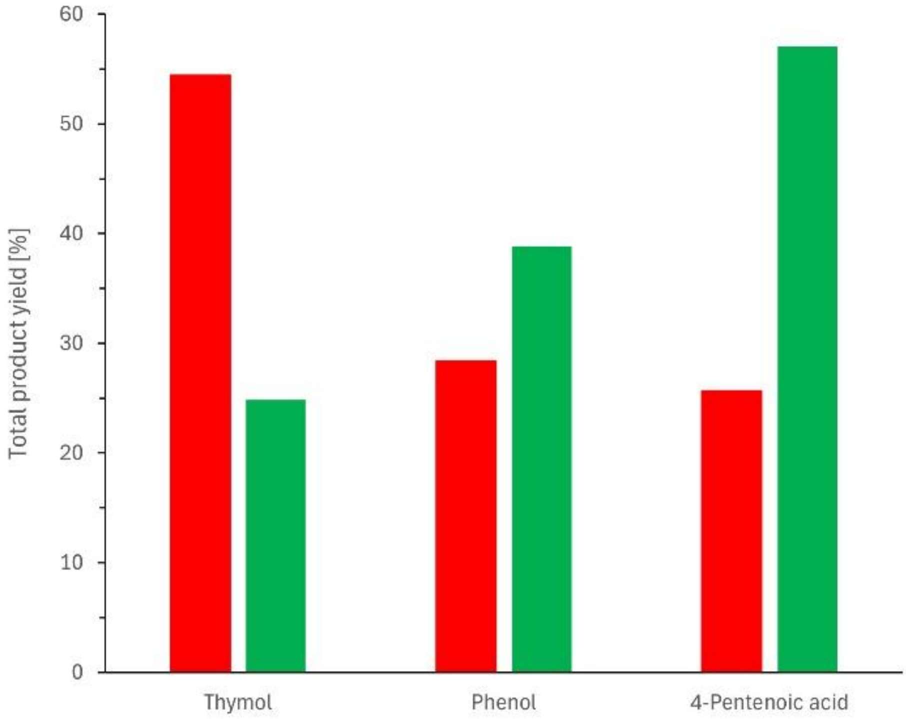

Figure S5: Comparison of total product formation in halogenation reactions using thymol, phenol, and 4-pentenoic acid. The bars in red show total product formation (%) in bioconversion reactions where only sodium formate was used to drive the reaction. The green bars show total product formation (%) in the bioconversion reactions where the reactions were started with the addition of Formate, but after two hours H_2O_2 was added instead.

Comparison of GC chromatograms with- and without presence of the biocatalyst (fusion protein)

To check if the oxidation of thioanisole is indeed catalyzed by the fusion protein and not by a chemical background reaction, experiments were performed adding thioanisole and H_2O_2 to the reaction mixture in absence of an enzymatic catalyst. In parallel, the oxidation of thioanisole in presence of a fusion protein catalyst was also performed. Figure S6 shows GC chromatograms of these reactions in absence of the fusion protein (S6, a), in presence of the fusion protein (S6, b) and the chromatogram at the start of the reaction (S6, c). A small product peak (retention time 6.9 minutes) can be observed in the reaction where the fusion protein is not present, but the amount of formed product is neglectable compared to the amount of product that is formed when the fusion protein is present.

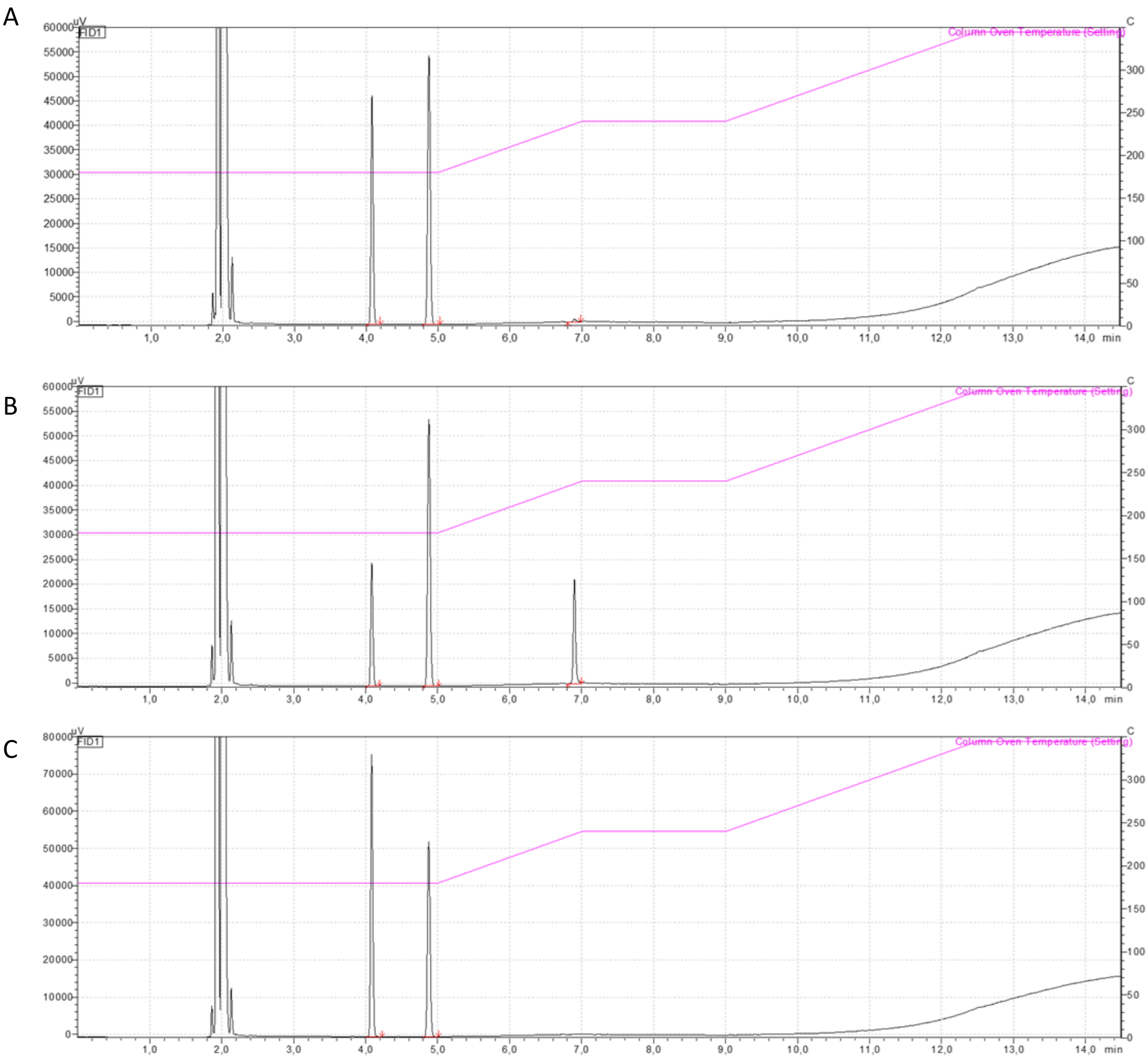

Figure S6: Comparison of product formation during thioanisole oxidation reaction in presence of the fusion protein (a) and without fusion protein (b) and the chromatogram at t=0 (c). Reaction conditions: 200 mM acetate buffer (pH 4.0), 10 v% cosolvent (ACN), 10 mM substrate, and 10 v% crude cell extract (corresponding to 0.5 U AoFOx activity for CE expression fusion protein C10fA). Reactions were incubated at 30 °C in an Eppendorf thermoshaker while shaking at 600 rpm. Reaction with the C10fA fusion protein were driven by adding formate (50 mM per two hours). To the negative control reaction with the EV construct, 2 mM H_2O_2 was added. Retention time thioanisole (substrate) = 4.1 minutes, retention time n-dodecane (internal standard) = 4.9 min, retention time methyl-phenyl-sulfide (product) = 6.9 minutes.

References

[1] G. Bonneau, A. A. M. Peru, A. L. Flourat, F. Allais, "Organic solvent- and catalyst-free Baeyer–Villiger oxidation of levoglucosenone and dihydrolevoglucosenone (Cyrene®): a sustainable route to (*S*)-γ-hydroxymethyl-α,β-butenolide and (*S*)-γ-hydroxymethyl-γ-butyrolactone" *Green Chem.* **2018**, *20*, 2455–2458.

[2] H. Mattes, C. Benezra, "Synthesis of a model hapten with cyclohexanediol and .alpha.-methylene-.gamma.-butyrolactone groups, a synthetic analog of poison ivy and tulipalin allergens connected with a carbon chain" *J. Org. Chem.* **1988**, *53*, 2732–2737.

[3] K. D. Belsare, A. J. Ruff, R. Martinez, A. V. Shivange, H. Mundhada, D. Holtmann, J. Schrader, U. Schwaneberg, "P-LinK: A Method for Generating Multicomponent Cytochrome P450 Fusions with Variable Linker Length" *BioTechniques* **2014**, *57*, 13–20.

[4] P. J. Bakkes, J. L. Riehm, T. Sagadin, A. Rühlmann, P. Schubert, S. Biemann, M. Girhard, M. C. Hutter, R. Bernhardt, V. B. Urlacher, "Engineering of versatile redox partner fusions that support monooxygenase activity of functionally diverse cytochrome P450s" *Sci. Rep.* **2017**, *7*, 9570.

[5] Y. Maeda, D. Doubayashi, M. Oki, H. Nose, A. Sakurai, K. Isa, Y. Fujii, H. Uchida, "Expression in *Escherichia coli* of an Unnamed Protein Gene from *Aspergillus oryzae* RIB40 and Cofactor Analyses of the Gene Product as Formate Oxidase" *Biosci. Biotechnol. Biochem.* **2009**, *73*, 2645–2649.

[6] E. F. Gérard, T. Mokkawes, L. O. Johannissen, J. Warwicker, R. R. Spiess, C. F. Blanford, S. Hay, D. J. Heyes, S. P. De Visser, "How Is Substrate Halogenation Triggered by the Vanadium Haloperoxidase from *Curvularia inaequalis*?" *ACS Catal.* **2023**, *13*, 8247–8261.
